# HNRNPA1-induced spliceopathy in a transgenic mouse model of myotonic dystrophy

**DOI:** 10.1073/pnas.1907297117

**Published:** 2020-02-21

**Authors:** Moyi Li, Yan Zhuang, Ranjan Batra, James D. Thomas, Mao Li, Curtis A. Nutter, Marina M. Scotti, Helmut A. Carter, Zhan Jun Wang, Xu-Sheng Huang, Chuan Qiang Pu, Maurice S. Swanson, Wei Xie

**Affiliations:** ^a^School of Life Science and Technology, The Key Laboratory of Developmental Genes and Human Disease, Southeast University, 210096 Nanjing, China;; ^b^Department of Molecular Genetics and Microbiology, Genetics Institute and the Center for NeuroGenetics, University of Florida, College of Medicine, Gainesville, FL 32610;; ^c^Jiangsu Co-innovation Center of Neuroregeneration, Nantong University, 226001 Nantong, China;; ^d^Department of Neurology, The General Hospital of Chinese People’s Liberation Army, 100853 Beijing, China;; ^e^Department of Neurology, Xuan Wu Hospital of Capital Medical University, 100053 Beijing, China

**Keywords:** microsatellite, splicing, HNRNPA1, MBNL1, CELF1

## Abstract

Studies on myotonic dystrophy type 1 (DM1) have led to the RNA-mediated disease model for hereditary disorders caused by noncoding microsatellite expansions. This model proposes that DM1 disease manifestations are caused by a reversion to fetal RNA processing patterns in adult tissues due to the expression of toxic CUG RNA expansions (CUG^exp^) leading to decreased muscleblind-like, but increased CUGBP1/ETR3-like factor 1 (CELF1), alternative splicing activities. Here, we test this model in vivo, using the mouse *HSA*^LR^ poly(CUG) model for DM1 and recombinant adeno-associated virus (rAAV)-mediated transduction of specific splicing factors. Surprisingly, systemic overexpression of HNRNPA1, not previously linked to DM1, also shifted DM1-relevant splicing targets to fetal isoforms, resulting in more severe muscle weakness/myopathy as early as 4 to 6 wk posttransduction, whereas rAAV controls were unaffected. Overexpression of HNRNPA1 promotes fetal exon inclusion of representative DM1-relevant splicing targets in differentiated myoblasts, and HITS-CLIP of rAAV-mycHnrnpa1-injected muscle revealed direct interactions of HNRNPA1 with these targets in vivo. Similar to CELF1, HNRNPA1 protein levels decrease during postnatal development, but are elevated in both regenerating mouse muscle and DM1 skeletal muscle. Our studies suggest that CUG^exp^ RNA triggers abnormal expression of multiple nuclear RNA binding proteins, including CELF1 and HNRNPA1, that antagonize MBNL activity to promote fetal splicing patterns.

## Significance

Myotonic dystrophy type 1 (DM1) is a model for RNA-mediated disease in microsatellite expansion disorders. DM1 is caused by CTG expansions (CTG^exp^) and expression of CUG^exp^ RNAs that sequester muscleblind-like (MBNL) proteins, while also triggering hyperphosphorylation of CUGBP1/ETR3-like factor 1 (CELF1). These proteins regulate developmental transitions in RNA processing, so DM1 is characterized by retention of fetal RNA processing patterns in adults. Although current evidence indicates that CELF1 is a specific antagonist of MBNL activity, this study reveals that another protein, HNRNPA1, is also downregulated during normal development but upregulated in DM1, where it also induces fetal splicing shifts. Thus, DM1 disease results from an imbalance in the expression of multiple RNA processing factors important for both proliferation and differentiation.

Microsatellite expansions in the noncoding regions of several human genes are associated with hereditary neurological diseases, including fragile X mental retardation (FRAXA) and myotonic dystrophy (DM) (1). In DM type 1 (DM1), the transcription of a CTG microsatellite expansion (CTG^exp^) in the 3′ untranslated region (3′ UTR) of the *DMPK* gene results in the expression of toxic CUG^exp^ RNA, which sequesters muscleblind-like (MBNL) proteins and blocks their splicing activity (2, 3). MBNL inhibition in DM1 tissues leads to a shift from adult to fetal splicing events for MBNL-targeted RNAs and results in tissue-specific disease manifestations, including muscle hyperexcitability (myotonia) and insulin resistance. In addition, CUG^exp^ RNA also activates protein kinase C (PKC), resulting in the hyperphosphorylation and elevated levels of CUGBP1/ETR3-like factor 1 (CELF1) in DM1 muscle and heart (4). In contrast to MBNL, CELF1 induces fetal splicing events in embryonic and early neonatal stages, and increased CELF1 levels in adult DM1 tissues causes a reversion to fetal isoforms. Thus, the current DM1 disease model is that coordinate MBNL sequestration and CELF1 accumulation caused by CUG^exp^ RNA expression synergistically promote aberrant fetal splicing patterns in DM1 (3, 5).

Support for both MBNL loss of function and CELF gain of function has come from mouse knockout and transgenic models (6–8). *Mbnl1* knockout adults develop muscle myotonia and myopathy, subcapsular dust-like cataracts, and alternative splicing changes characteristic of DM1, while compound loss of MBNL1 and MBNL2 is required for the onset of severe muscle wasting (6, 9). In contrast, *Mbnl2* knockouts recapitulate CNS features of DM1, including hypersomnia and learning/memory deficits, but additional MBNL1 loss is required for MAPT/tau missplicing (9, 10). *Mbnl3* is expressed during muscle regeneration, and loss of its major protein isoform delays adult muscle regeneration in mice (11). Transgenic CELF1 overexpression mice are characterized by impaired myogenesis, centralized myonuclei, and muscle degeneration similar to transgenic mice expressing an inducible CTG^exp^ in skeletal muscle (EpA960/HSA-Cre- ER^T2^) (8, 12, 13). In addition to CELF and MBNL proteins, other factors have also been proposed to play a role in disease onset. For example, levels of the double-stranded RNA-binding protein Staufen1 (STAU1) also increase in DM1 tissues, and STAU1 overexpression promotes a splicing shift to adult isoforms, suggesting it may be an effective DM1 disease modifier (14, 15).

Here, we demonstrate that overexpression of HNRNPA1, similar to CELF1, has the opposite effect as STAU1 and triggers DM1 disease muscle pathology while shifting the splicing of DM1 RNA targets to an earlier developmental pattern (8). Moreover, HNRNPA1 expression declines during normal postnatal skeletal muscle development, but is up-regulated during muscle regeneration and in DM1 muscle. Our results suggest that CUG^exp^ RNA expression impacts the levels of multiple nuclear RNA binding proteins, including members of the hnRNP family, that are important for cell proliferation, resulting in a reversal to an early developmental RNA processing program.

## Results

### Reduced Lifespan and Muscle Pathology Following HNRNPA1 Overexpression in a Mouse DM1 Model.

Previously, we proposed a gene therapy strategy for DM1 that involved MBNL overexpression, leading to an increase in the nonsequestered nuclear pool of MBNL protein and reversal of DM1-associated RNA missplicing and pathology. To test this therapeutic approach, we demonstrated that MBNL1 was overexpressed following intramuscular (i.m.) injection of rAAV2/1-mycMbnl1 virus, and this led to the reversal of myotonia and missplicing of DM1 target RNAs in the *HSA*^LR^ poly(CUG) model for DM1 (16). In this study, our initial objective was to determine if systemic overexpression of MBNL proteins would have a similar effect, and we selected rAAV2/9 as the viral vector, since it has been shown to more effectively transduce a wider array of tissues using either i.p. or i.v. injections (17, 18). Furthermore, MBNL2 (rAAV2/9-mycMbnl2) was chosen for overexpression, since this paralog compensates for loss of MBNL1 due to sequestration by CUG^exp^ RNA in skeletal muscle and is the major MBNL protein expressed in the brain (9). As a control for overexpression of an unrelated nuclear RNA binding protein, the effects of hnRNP A1 (HNRNPA1) overexpression were also characterized by systemic expression of rAAV2/9-mycHnrnpA1. In addition, rAAV2/9-mycCelf1 was generated for CELF1 systemic overexpression, since it is a well-characterized DM1-related RNA missplicing effector.

**Fig. 1.**
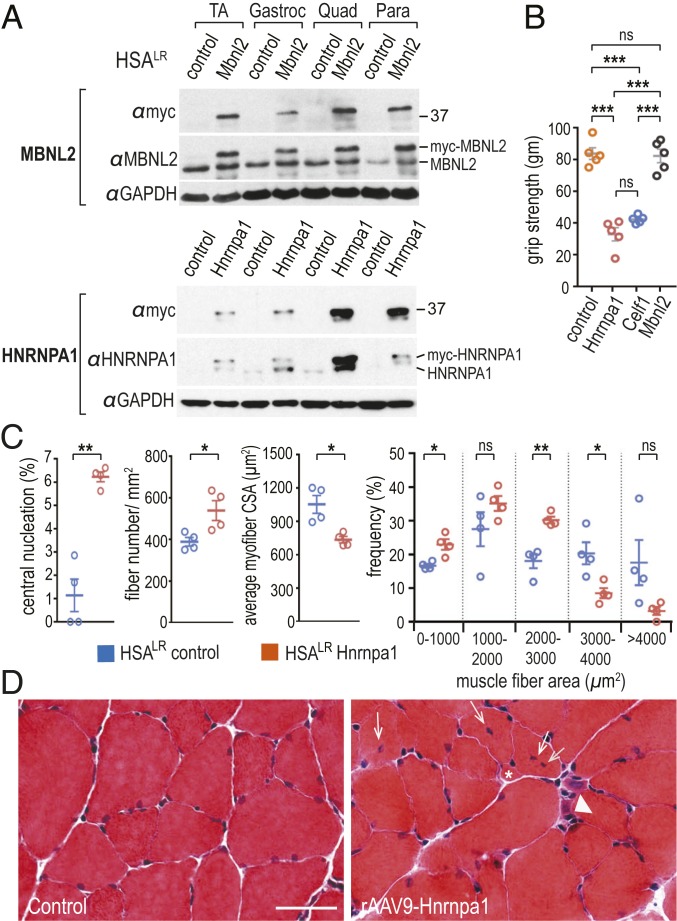
AAV9-mediated systemic overexpression of HNRNPA1 in a mouse model of DM1 leads to disease-associated pathology. (*A*) *HSA*^LR^ P0-P2 mice (*n* = 4 each) were injected (i.v.) with PBS (control), AAV9/Mbnl2 (Mbnl2), or AAV9/HnrnpA1 (Hnrnpa1) and protein levels were assessed by immunoblotting using antibodies against both endogenous (MBNL2, HNRNPA1) and exogenous (myc-MBNL2, myc-HNRNPA1) proteins in tibialis anterior (TA), gastrocnemius (Gastroc), quadriceps (Quad) and paraspinal (Para) muscles. GAPDH served as the loading control. (*B*) AAV9-mediated overexpression of either CELF1 (AAV9-Celf1, *n* = 5) or HNRNPA1 (AAV9-Hnrnpa1 *n* = 5) led to reductions in grip strength compared with PBS (*n* = 5), GFP (AAV9-GFP, *n* = 5), or Mbnl2-injected (AAV9-Mbnl2, *n* = 5) mice. (*C*) Statistical analysis and (*D*) representative muscle cross-sections indicated an earlier onset of DM1-relevant myopathic changes including centralized myonuclei (white arrows), atrophic (white arrowhead) myofibers, and split (asterisk) fibers in HNRNPA1 (rAAV9-Hnrnpa1) overexpression quadriceps. *P* values were calculated using a one-way ANOVA with Tukey’s HSD post hoc test or an unpaired two-tailed Student’s *t* test. Data are SEM and significant. *P < 0.05; **P < 0.01; ***P < 0.001; ns, not significant. (Scale bar, 50 µm.)

Following injections into the temporal vein of *HSA*^LR^ neonates (postnatal days 0 to 2, P0 to P2), mice were sacrificed at 4 to 6 wk of age, and four muscle groups (tibialis anterior, gastrocnemius, quadriceps, paraspinals) were analyzed for myc-Mbnl2 as well as endogenous MBNL2 protein levels (Fig. 1*A*). Similar levels of endogenous MBNL2 were observed by immunoblot analysis in control (PBS-injected) versus rAAV2/9-mycMbnl2/40-injected mice, while myc-MBNL2 was only detectable in the latter muscles. In contrast, endogenous HNRNPA1 was either undetectable or detectable at very low levels in control *HSA*^LR^ adult muscles, presumably due to the relatively low proliferative state and transcriptional activity of differentiated skeletal muscle, while AAV-mediated overexpression of HNRNPA1 or CELF1 varied considerably between different muscles, with quadriceps displaying the highest level (Fig. 1*A* and *SI Appendix*, Fig. S1). Surprisingly, myc-HnrnpA1 overexpression reduced the maximum lifespan of *HSA*^LR^ mice to ∼6 wk of age (*SI Appendix*, Fig. S2*A*) and resulted in muscle weakness (Fig. 1*B*), as well as moderate muscle pathology (increased centralized nuclei, myofiber atrophy, and sporadical ring fibers; Fig. 1 *C* and *D* and *SI Appendix*, Fig. S2*B*) in 4- to 6-wk-old mice, while rAAV2/9-GFP and rAAV2/9-mycMbnl2 were similar to uninjected *HSA*^LR^ mice (7). Systemic HNRNPA1 overexpression also resulted in aberrant motility (Movies S1–S3), although comparison of injected versus uninjected neuromuscular junctions did not show obvious abnormalities caused by HNRNPA1 overexpression (*SI Appendix*, Fig. S3).

### HNRNPA1 Induces DM1-Associated Fetal Splicing Patterns.

**Fig. 2.**
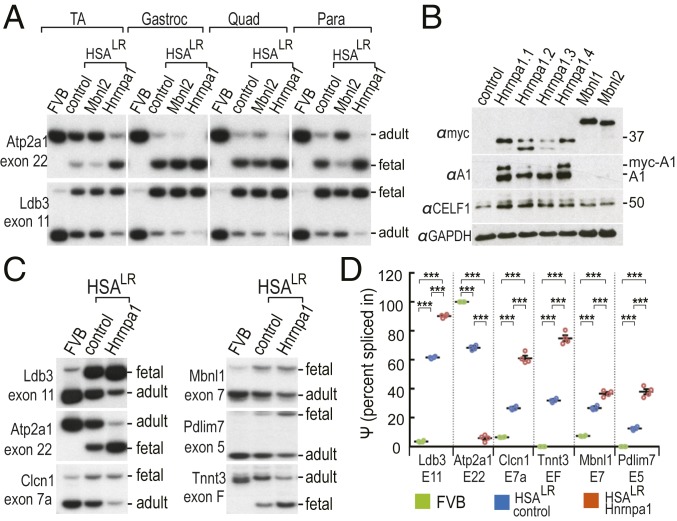
Increased expression of HNRNPA1 in skeletal muscles promotes fetal splicing patterns for DM1 targets. (*A*) Systemic HNRNPA1 overexpression exacerbated, while MBNL2 partially reversed, DM1 fetal splicing patterns in *HSA*^LR^ muscles. RT-PCR analysis of Atp2a1 exon 22 and Ldb3 exon 11 splicing patterns were used as representative DM1 targets and positions of the fetal and adult exon splicing RT-PCR products are indicated (*Right*). Alternative cassette exon splicing was determined in wild-type (FVB) and *HSA*^LR^ mice without injection (control) or following AAV9-Mbnl2 (Mbnl2) or AAV9-HnrnpA1 *HSA*^LR^ injections (*n* = 4 each). (*B*) Immunoblotting for myc-tagged HNRNPA1 (αmyc), HNRNPA1 (αA1), CELF1 (αCELF1), and the loading control GAPDH (αGAPDH) following direct i.m. injections of AAV9-mycHnrnpa1 (*n* = 4 mice Hnrnp1.1-1.4), AAV9-mycMbnl1, or AAV9-mycMbnl2. The positions of myc-HNRNPA1 (myc-A1) and endogenous HNRNPA1 (A1) are indicated. (*C*) RT-PCR splicing analysis of six DM1 targeted gene transcripts demonstrated that HNRNPA1 overexpression in *HSA*^LR^ TA promoted fetal splicing events. The wild-type splicing pattern for each gene is shown in the FVB lanes. (*D*) Fetal exon inclusion was determined using percentage spliced in (Ψ; *n* = 4). *P* values were calculated using a one-way ANOVA with Tukey’s HSD post hoc test. Data are SEM and significant. ***P < 0.001.

To assess the effects of rAAV-mediated RNA binding protein overexpression on alternative splicing regulation, RT-PCR of RNA targets misspliced in DM1 muscle was performed. In paraspinal muscles, MBNL2 overexpression after rAAV2/9-mycMbnl2 transduction shifted the splicing of DM1 target pre-mRNAs, including Atp2a1/Serca1 and Ldb3/Cypher, to a more adult pattern, while splicing changes in other muscle groups were either unchanged or more modest (Fig. 2*A*). This result may reflect variations in *HSA*^LR^ transgene expression levels or Mbnl2 subcellular localization in these muscles from relatively young (4 wk of age) mice. Contrary to MBNL2, HNRNPA1 overexpression led to a striking shift of these RNAs to a more fetal pattern in all muscles examined, similar to the splicing changes observed previously in CELF1 overexpression mice (8). These results suggested that in addition to CELF1, the splicing activities of other nuclear RNA-binding proteins might be affected by expression of CUG^exp^ RNAs.

To confirm that these splicing shifts were not the result of systemic pathology and compromised lifespan following neonatal systemic delivery of AAV, DM1-relevant splicing shifts were subsequently evaluated in adult skeletal muscle following direct i.m. injections. The levels of HNRNPA1 overexpression were more consistent following i.m. versus systemic injections, although a shorter myc-HNRNPA1 isoform was detectable in some muscle samples (Fig. 2*B*, αmyc lanes Hnrnpa1.2 and 1.3) and the levels of proteins comigrating with the endogenous 34-kDa HNRNPA1 protein also substantially increased (Fig. 2*B**,* αHNRNPA1 lanes). Up-regulation of endogenous CELF1 has also been observed in mice overexpressing His-CELF1 (12). In agreement with the systemic overexpression study, HNRNPA1 overexpression following i.m. injections resulted in a shift to the fetal pattern for DM1 splicing targets including Ldb3 exon 11, Atp2a1 exon 22, Clcn1 exon 7a, Mbnl1 exon 7, Pdlim exon 5, and Tnnt3 exon F (Fig. 2 *C* and *D*). Previous work has shown that CELF1 protein levels in skeletal muscle are not significantly different between wild-type and *HSA*^LR^ mice (19) and CELF1 levels were relatively consistent following overexpression of MBNL1 or MBNL2, although AAV/HNRNPA1 transduction induced a modest up-regulation of CELF1 in injected muscle tissue. To exclude the possibility that these splicing events reflected secondary effects from HNRNPA1-induced CELF1 up-regulation, we compared these DM1 targets with CELF1 splicing targets in CELF1-overexpressing transgenic mice (8). Importantly, Clcn1 exon 7a, Mbnl1 exon 5, and Tnnt3 exon F are not affected by CELF1 overexpression (8) while they were altered by HNRNPA1 overexpression in our study, indicating that the splicing shifts following HNRNPA1 overexpression were not due to CELF1 up-regulation.

**Fig. 3.**
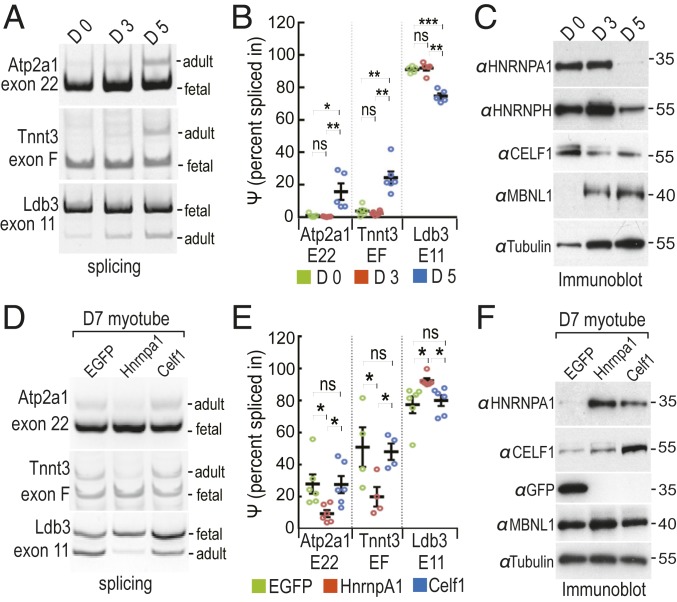
Alternative splicing assay in myoblast/myotube cultures confirms DM1 fetal exon splicing is promoted by HNRNPA1. (*A*) Splicing patterns of three endogenous DM1 targets (Atp2a1 exon 22, Tnnt3 exon F, and Ldb3 exon 11) in myoblasts at day 0 (D0), day 3 (D3), and day 5 (D5) after differentiation. (*B*) Endogenous DM1 exon splicing in *A* was determined using percentage spliced in (Ψ) (*n* = 5). (*C*) Western blot analysis of four RNA binding proteins (HNRNPA1, HNRNPH, CELF1, and MBNL1) in myoblasts at D0, D3, and D5. (*D*) Effects of lenti-induced overexpression HnrnpA1 and Celf1 on endogenous DM1 targets in differentiated myofibers harvested at day 7 (D7). AAV9/EGFP is used as a negative control. (*E*) DM1 exon splicing in *D* was determined using Ψ (*n* = 4). (*F*) Immunoblot of four RNA binding proteins (HNRNPA1, HNRNPH, CELF1, and MBNL1) in AAV9/EGFP (EGFP), AAV9/HnRNPA1 (HnrnpA1), and AAV9/CELF1 (Celf1) transduced myoblasts harvested at D7. *P* values were calculated using a one-way ANOVA (or RM one-way ANOVA) with Tukey’s HSD post hoc test. Data are SEM and significant. *P < 0.05; **P < 0.01; ***P < 0.001; ns, not significant.

To further investigate the effects of HNRNPA1 on DM1 splicing targets, the splicing activity of HNRNPA1 and CELF1 proteins was also examined using mouse primary myoblasts transduced with lentiviruses overexpressing HNRNPA1 or CELF1 either prior to (day 0, D0) or after induction of myogenic differentiation into myotubes for days 3 to 7 (D3, D5, D7), D3-7. The splicing patterns of the DM1 representative splicing targets Atp2a1 exon 22, Ldb3 exon 11, and Tnnt3 fetal exon showed a differentiation time-dependent increase in the adult to fetal isoform ratio (Fig. 3 *A* and *B*) together with a coordinate decrease in HNRNPA1, HNRNPH, and CELF1, as well as increase in MBNL1, protein levels (Fig. 3*C*). Similar to *HSA*^LR^ muscle, HNRNPA1 overexpression in differentiated myotubes resulted in significant shifts in Atp2a exon22, Ldb3 exon 11, and Tnnt3 F exon splicing to a more fetal pattern, whereas CELF1 overexpression did not (Fig. 3 *D*–*F*). Overall, these results indicated that elevated levels of HNRNPA1 led to a reversion of the splicing program to a fetal pattern characteristic of DM1 muscle. To demonstrate that these splicing alterations were a direct effect of HNRNPA1 binding to regulatory regions proximal to MBNL1-regulated exons, HITS-CLIP/CLIP-seq analysis was performed.

### Direct Binding of HNRNPA1 to MBNL-Regulated Exons.

**Fig. 4.**
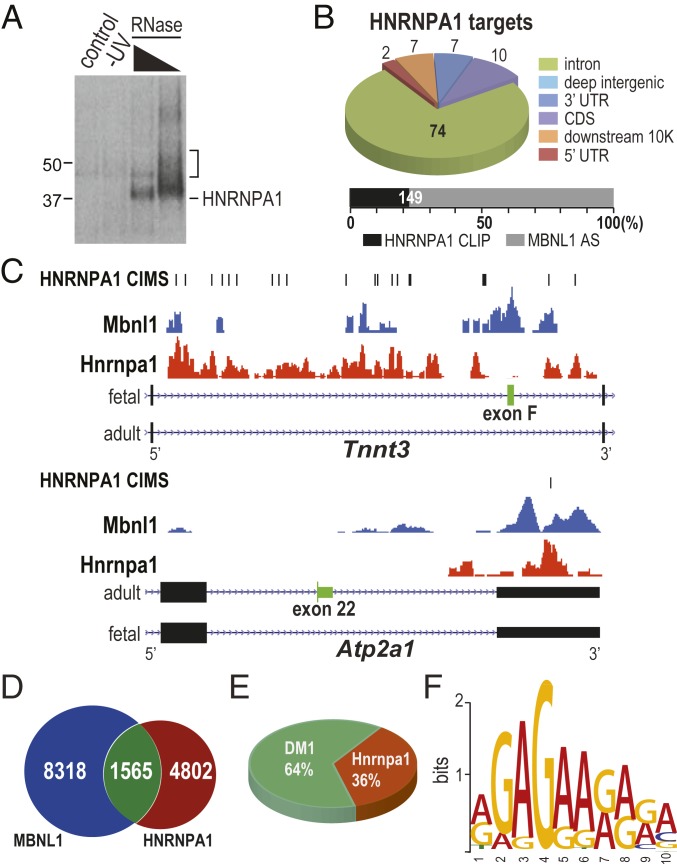
HITS-CLIP confirms that MBNL1 and HNRNPA1 proteins bind a set of overlapping RNA targets. HNRNPA1 binds directly to MBNL-regulated exons. (*A*) Protein gel autoradiograph of HNRNPA1-RNA ^32^P-labeled complexes after cross-linking, RNase A digestion and immunopurification with anti-HNRNPA1 mAb 4B10 from myc-HNRNPA1-injected *HSA*^LR^ quadriceps. PBS-injected (control) and uncrosslinked (-UV) controls are also shown. (*B*) Pie chart of HNRNPA1-CLIP tag distribution. (*Lower*) A stacked bar chart of the fraction of total MBNL1-regulated alternatively spliced exons bound by HNRNPA1 (±500 bp): MBNL1-regulated AS events (gray, *n* = 666) (45), AS events covered by HNRNPA1 CLIP peaks (black, *n* = 149). (*C*) Wiggle tracks of HNRNPA1 CLIP tag density for *Tnnt3*, and *Atp2a1* for MBNL-regulated alternative exons. The MBNL1 CLIP-seq FVB WT quadriceps muscle dataset has been reported previously (46). (*D*) Venn diagram of overlap between HNRNPA1 binding sites and MBNL1 binding sites in FVB quadriceps. (*E*) Pie chart of overlap between HNRNPA1-CLIP tags and DM1 relative AS events in *HSA*^LR^ transgenic mice. (*F*) De novo motif search using sequences from top 500 CLIP clusters and MEME.

To determine if HNRNPA1 bound in the vicinity of MBNL-regulated alternative cassette exons, HITS-CLIP transcriptome-wide analysis was performed on *HSA*^LR^ quadriceps muscle injected with rAAV2/9-HnrnpA1 (Fig. 4*A*). The genomic distribution of CLIP peaks showed that the majority (74%) of the HNRNPA1 binding sites were intronic, consistent with the previously established role of this protein in splicing regulation (Fig. 4*B* and Dataset S1A) (20). Within the flanking introns of some regulated alternative cassette exons, HNRNPA1 showed more upstream binding for inclusion, and more downstream binding for exclusion, of MBNL-regulated cassettes (Fig. 4*C*, *Tnnt3* and *Atp2a1*, respectively; Dataset S1 B and C). Furthermore, considerable overlap was observed between MBNL1-regulated exons identified by exon junction microarray analysis and proximal binding sites for HNRNPA1 (Fig. 4*D*). Further comparison of HNRNPA1 binding events to the top 50 alternative exons/introns in *HSA*^LR^ muscle identified 18 of 50 alternative splicing sites bound by HNRNPA1, including key events that may be used as clinical biomarkers for DM1 (Fig. 4*E* and Dataset S1D). Previous SELEX experiments and CLIP-seq analyses have shown preferential binding of HNRNPA1 to UAGGGA/U and UAGU sequence elements, respectively (20, 21), while in skeletal muscle, HNRNPA1 preferentially crosslinked to AG-rich sequences (Fig. 4*F*). Therefore, HNRNPA1 binds directly, and acts antagonistically, to MBNL1 in skeletal muscle and promotes both inclusion of fetal and skipping of adult exons previously implicated in DM1 spliceopathy.

### Down-Regulation of HNRNPA1 RNA and Protein during Muscle Development.

**Fig. 5.**
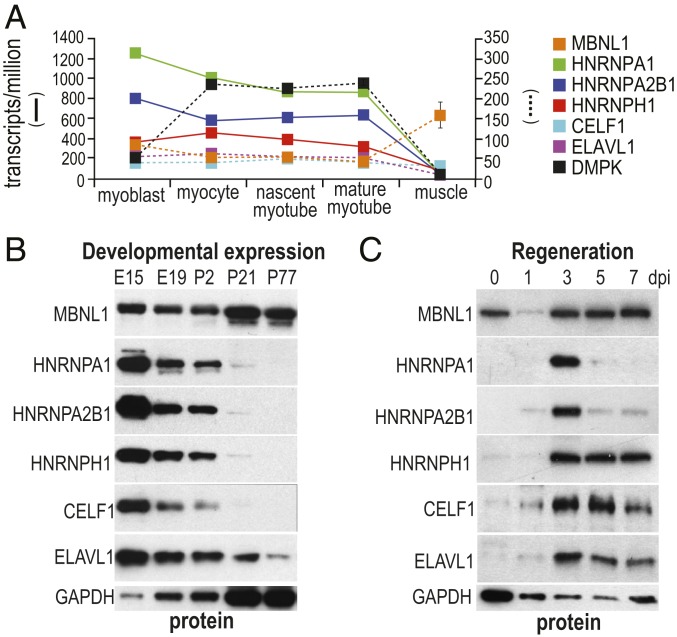
HNRNPA1 expression declines during mouse postnatal muscle development. (*A*) Human HNRNPA1 RNA levels decline as muscle precursor cells differentiate into mature skeletal muscle. Previously reported RNA-seq datasets were analyzed, including myoblasts, myocytes, nascent myotubes, and mature myotubes (22) and mature skeletal muscle (http://www.dmseq.org/; *n* = 3 for each sample). TPM (transcripts per million) values are shown for interested proteins. (*B*) Developmental expression of MBNL1 and hnRNP proteins (HNRNPA1, HNRNPA2/B1, HNRNPH), as well as CELF1 and ELAVL1, show opposite expression trends during muscle development from embryonic day (E)15 to postnatal day (P)77. (*C*) Immunoblot showing that HNRNPA1 is only transiently expressed at day 3 postinjection (dpi) during muscle regeneration after Notexin-induced muscle ablation and regeneration compared with other RNA binding proteins.

HnRNPs are abundant and multifunctional nuclear RNA binding proteins in proliferating cultured cells. Since HNRNPA1 overexpression promoted fetal splicing, the expression of hnRNP genes implicated in several microsatellite expansion diseases, including amyotrophic lateral sclerosis and frontotemporal dementia and DM1, was examined using existing RNA-seq datasets of human muscle differentiation (22) and compared with control skeletal muscle from the DM deep sequencing data repository (http://dmseq.org/). In contrast to MBNL1, the expression levels of HNRNPA1, HNRNPA2B1, HNRNPH1, CELF1, and ELAVL1 RNAs all decrease during normal myogenic differentiation in vitro from myoblasts to mature myotubes (Fig. 5*A* and Dataset S1E). In wild-type adult skeletal muscle, these RNAs are expressed at low levels, and the corresponding protein levels during mouse skeletal muscle development confirmed that all these RBPs declined during muscle development, with the exception of the MBNL1 protein, in agreement with our data for protein accumulation patterns of these RBPs in differentiating myoblasts (Fig. 3*C*), and previous studies on MBNL1 and CELF1 expression during embryonic and postnatal skeletal and heart muscle development (Fig. 5*B*) (23, 24). To address the question of how these protein levels vary during adult muscle regeneration, tibialis anterior muscles were injected with notexin, a snake venom phospholipase A2 that triggers sarcolemma disruption leading to transient myofiber loss. Previously, we showed that mature myofibers are mostly lost at day 1 postinjection, and proliferating myoblasts are elevated by day 3 following injection (11).Consistent with our previous observations for MBNL1 RNA expression patterns during notexin-induced muscle regeneration (11), MBNL1 protein was expressed throughout the regeneration course only with a transient decrease at day 1 following injection (Fig. 5*C*). In contrast to MBNL1, HNRNPA1, HNRNPA2B1, HNRNPH, CELF1, and ELAVL1 protein levels were relatively low until day 3 postinjection. Interestingly, HNRNPA1 upregulation was only detected at the early regeneration stage (day 3 postinjection), whereas HNRNPH, CELF1, and ELAVL1 proteins remained through day 7, when myofibers have regenerated (Fig. 5*C*). We conclude that *Hnrnpa1*/*HNRNPA1* genes are down-regulated during normal development, and only transiently expressed during adult regeneration, of skeletal muscle, while DM1 muscle recapitulates the fetal HNRNPA1 expression pattern. Next, we compared the expression of HNRNPA1 and CELF1 in DM1 muscle.

### HNRNPA1 Protein Is Elevated in DM1 Biopsied Muscle.

**Fig. 6.**
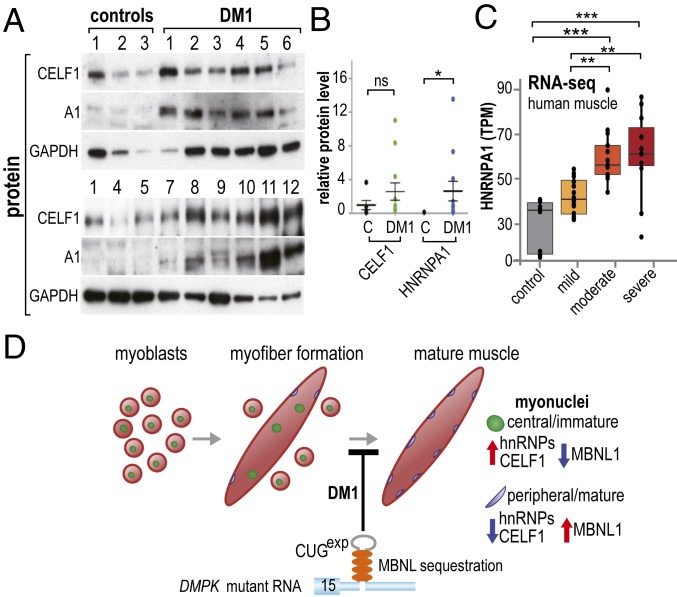
Up-regulation of both HNRNPA1 and CELF1 in DM1 muscle. (*A*) Immunoblots of control (*n* = 5) and DM1 (*n* = 12) muscle biopsy samples (GAPDH, loading control). (*B*) Quantitative analysis of relative protein levels (CELF/GAPDH, HNRNPA1/GAPDH) between control (*C*) and DM1 muscle biopsies. Note the very low level of HNRNPA1 in control adult muscles. *P* values were calculated using unpaired one-tailed Student’s *t* test. Data are SEM and significant. *P = 0.0268. (*C*) Box plots of HNRNPA1 mRNA levels (transcripts per million, TPM) in tibialis anterior muscle biopsies from control or DM1 patients. DM1 patients were classified as mild, moderate, or severe, as previously described (47). Patient RNA-seq data were acquired from the Myotonic Dystrophy Deep Sequencing Data Repository (http://www.dmseq.org/). *P* values were calculated using a one-way ANOVA with Tukey’s HSD post hoc test. Data are SEM and significant. **P < 0.01; ***P < 0.001. (*D*) Model for RBP misregulation in DM1 pathogenesis. Normally, myoblasts (*Left*, red circles with green central/immature nuclei) fuse to form immature myofibers (*Middle*, red ellipsoid) followed by nuclear migration to the subsarcolemmal region resulting in mature myofibers (*Right*, blue peripheral/mature nuclei). In DM1, the CUG expansion (gray hairpin in DMPK 3′ UTR) inhibits this normal differentiation process so DM1 myofibers are characterized by centralized myonuclei. At the molecular level, MBNL (orange ovals) splicing activity is down-regulated (blue arrow) due to sequestration by CUG^exp^ RNAs, while hnRNPs and CELF1 are up-regulated (red arrow) either at the transcriptional (HNRNPA1) or posttranslational (CELF1) levels, leading to coordinate misregulation of these RNA splicing factors and DM1 spliceopathy.

Initially, we attempted to determine HNRNPA1 protein levels using control versus DM1 autopsy muscles, but immunoblotting either failed to detect HNRNPA1 or detected polypeptides migrating below the HNRNPA1 34-kDa full-length protein. To avoid the potential problem of postmortem protein degradation, biopsied samples were obtained from both control and DM1 muscles (Dataset S1F). In DM1 skeletal and heart muscle, CUG^exp^ RNAs activate PKC signaling and lead to hyperphosphorylation and increased levels of CELF1 (4). Although we also detected an increase in CELF1 in DM1 biopsied muscles compared with controls (Fig. 6 *A* and *B*), HNRNPA1 levels significantly increased in DM1. RNA-seq data (DMseq.org) indicated a similar increase in HNRNPA1 transcripts in DM1 biopsies, and DM1 disease severity corresponded with increased *HNRNPA1* transcription (Fig. 6*C*). To test if other hnRNPs have similar effects on DM1 targets, protein expression levels for two additional hnRNPs, HNRNPH1 and HNRNPA2B1, were examined in DM1 biopsies, but the expression levels of these hnRNPs were not significantly different from controls (*SI Appendix*, Fig. S4 *A* and *B*). Moreover, an increase in fetal splicing shifts of representative muscle DM1 targets was not observed in AAV/Hhnrph1-transduced myofibers (*SI Appendix*, Fig. S4*C*), and MBNL1 levels were not markedly perturbed in DM1 biopsies or HNRNPA1/CELF1-overexpressed myofibers (*SI Appendix*, Fig. S4 *A*, *B*, and *D*). Cumulatively, these data demonstrate that CELF1 up-regulation is not a unique diagnostic marker of DM1 muscle, and that this disease also induces an increase in HNRNPA1, and possibly other nuclear RNA binding proteins, that modulate the effects of MBNL sequestration and DM1-induced spliceopathy.

## Discussion

During a study designed to determine if systemic MBNL2 overexpression in neonatal mice would block disease progression in the *HSA*^LR^ polyCUG model of DM1, we also tested for nonspecific effects of an unrelated RBP, HNRNPA1. Surprisingly, HNRNPA1 recapitulated both the physiological and molecular effects associated with CELF1 overexpression, including reduced lifespan and muscle strength, muscle histopathology, and the persistence of specific DM1-relevant splicing patterns in adult tissues (8). Splicing assays using differentiated primary myoblasts confirmed that elevated HNRNPA1 protein levels reproduced DM1 splicing patterns, while HITS-CLIP of *HSA*^LR^ muscle overexpressing HNRNPA1 revealed that >30% of HNRNPA1 targets overlapped with MBNL1 target RNAs. The expression of multiple hnRNP genes declined, while MBNL1 increased, during late fetal and early postnatal skeletal muscle development and HNRNPA1 was only transiently overexpressed during adult muscle regeneration. Interestingly, DM1 muscle showed elevated HNRNPA1 protein levels. It is important to note that the increase in HNRNPA1 protein levels was only observed in biopsy DM1 muscle, since immunoblotting of autopsy muscle with anti-HNRNPA1 antibodies consistently failed to detect the full-length protein, likely due to postmortem proteolysis. Indeed, HNRNPA1 was originally characterized as the eukaryotic helix-unwinding single-stranded DNA binding protein UP1, a 24-kDa proteolytic fragment that contains two N-terminal RNA recognition motifs, but is missing the glycine-rich C terminus (25–27). On the basis of these results, we conclude that (CUG)_n_ repeat expansions trigger the overexpression of multiple RBPs, including CELF1 and HNRNPA1, that cumulatively drive fetal splicing events in DM1 adult tissues.

Although HNRNPA1 functions include transcription elongation (28), pri-miR processing (29, 30), mRNA translation, and telomere maintenance (31, 32), its role in pre-mRNA splicing has been the most thoroughly characterized (21, 33–35). In general, HNRNPA1 binds to exonic and intronic splicing silencers to repress exon inclusion, but it also activates inclusion of some exons and is involved in proofreading 3′ splice site recognition by U2AF (21, 34, 36, 37). Our results extend these prior observations by demonstrating that developmental splicing patterns are also affected by manipulating HNRNPA1 protein levels in vivo. Similar to CELF1, HNRNPA1 acts antagonistically to MBNL1 in skeletal muscle and promotes the inclusion of fetal exons, but the exclusion of adult exons, for a specific set of developmentally regulated genes. This developmental splicing activity agrees with prior studies that have shown that HNRNPA1 is highly expressed in proliferating cells and a wide range of cancers, but is either undetectable or present at much lower levels in most normal differentiated tissues (38, 39).

The demonstration that increased expression of HNRNPA1 recapitulates DM1 manifestations and splicing defects adds to a growing list of diseases attributed to HNRNPA1 misexpression and mutations, including Alzheimer’s disease (AD) and amyotrophic lateral sclerosis (40). In AD, HNRNPA1, in conjunction with SRSF2, regulates the splicing of APP exon 7, with HNRNPA1 acting as a repressor of this alternative cassette (41). Relevant to this study, MBNL2 functions antagonistically as an activator of this splicing event. MBNL sequestration by CUG expansion RNAs in DM1 results in enhanced APP exon 7 skipping (2), while HNRNPA1 knockdown leads to expression of full-length APP (+exon 7), which has been suggested to lead to increases in Aβ peptide secretion and amyloid plaques. Microarray analysis of control versus AD entorhinal cortex, which is severely affected in AD, shows striking increases in alternative exon inclusion and significant decreases in hnRNP A/B levels in AD (42). Several hereditary diseases have also been linked to HNRNPA1 missense mutations, including multisystem proteinopathy and amyotrophic lateral sclerosis. These mutations are located in the HNRNPA1 C-terminal region, which contains a prion-like domain that confers unusual physical properties to this protein, including the ability to phase separate into liquid droplets (43, 44), and may result in HNRNPA1 loss of function although the effects on splicing regulation have not been evaluated. In light of the observations reported here, further studies on HNRNPA1 functions in embryonic versus postnatal tissues are clearly warranted and may reveal additional regulatory functions of the hnRNP A/B family.

## Materials and Methods

Detailed information on DM1 biopsies, animal handling, viral preparation and administration, immunoblotting, muscle functional analysis and histology, primary myoblast culturing and lentiviral transduction, splicing analysis, CLIP-seq and RNA-seq, and muscle injury/regeneration are provided in *SI Appendix*, *Materials and Methods*.

### Data Availability Statement.

All data obtained for this study are presented within the main text, *SI Appendix*, and Dataset S1.

## Supplementary Material

Supplementary File

Supplementary File

Supplementary File

Supplementary File

Supplementary File

## References

[1] Iyer R. R., Pluciennik A., Napierala M., Wells R. D., DNA triplet repeat expansion and mismatch repair. Annu. Rev. Biochem. 84, 199–226 (2015). 25580529 10.1146/annurev-biochem-060614-034010 PMC4845744

[2] Goodwin M., MBNL sequestration by toxic RNAs and RNA misprocessing in the myotonic dystrophy brain. Cell Rep. 12, 1159–1168 (2015). 26257173 10.1016/j.celrep.2015.07.029 PMC4545389

[3] Echeverria G. V., Cooper T. A., RNA-binding proteins in microsatellite expansion disorders: Mediators of RNA toxicity. Brain Res. 1462, 100–111 (2012). 22405728 10.1016/j.brainres.2012.02.030 PMC3372679

[4] Kuyumcu-Martinez N. M., Wang G. S., Cooper T. A., Increased steady-state levels of CUGBP1 in myotonic dystrophy 1 are due to PKC-mediated hyperphosphorylation. Mol. Cell 28, 68–78 (2007). 17936705 10.1016/j.molcel.2007.07.027 PMC2083558

[5] Scotti M. M., Swanson M. S., RNA mis-splicing in disease. Nat. Rev. Genet. 17, 19–32 (2016). 26593421 10.1038/nrg.2015.3 PMC5993438

[6] Kanadia R. N., A muscleblind knockout model for myotonic dystrophy. Science 302, 1978–1980 (2003). 14671308 10.1126/science.1088583

[7] Mankodi A., Myotonic dystrophy in transgenic mice expressing an expanded CUG repeat. Science 289, 1769–1773 (2000). 10976074 10.1126/science.289.5485.1769

[8] Ward A. J., Rimer M., Killian J. M., Dowling J. J., Cooper T. A., CUGBP1 overexpression in mouse skeletal muscle reproduces features of myotonic dystrophy type 1. Hum. Mol. Genet. 19, 3614–3622 (2010). 20603324 10.1093/hmg/ddq277 PMC2928132

[9] Lee K. Y., Compound loss of muscleblind-like function in myotonic dystrophy. EMBO Mol. Med. 5, 1887–1900 (2013). 24293317 10.1002/emmm.201303275 PMC3914532

[10] Charizanis K., Muscleblind-like 2-mediated alternative splicing in the developing brain and dysregulation in myotonic dystrophy. Neuron 75, 437–450 (2012). 22884328 10.1016/j.neuron.2012.05.029 PMC3418517

[11] Poulos M. G., Progressive impairment of muscle regeneration in muscleblind-like 3 isoform knockout mice. Hum. Mol. Genet. 22, 3547–3558 (2013). 23660517 10.1093/hmg/ddt209 PMC3736872

[12] Timchenko N. A., Overexpression of CUG triplet repeat-binding protein, CUGBP1, in mice inhibits myogenesis. J. Biol. Chem. 279, 13129–13139 (2004). 14722059 10.1074/jbc.M312923200

[13] Orengo J. P., Expanded CTG repeats within the DMPK 3′ UTR causes severe skeletal muscle wasting in an inducible mouse model for myotonic dystrophy. Proc. Natl. Acad. Sci. U.S.A. 105, 2646–2651 (2008). 18272483 10.1073/pnas.0708519105 PMC2268190

[14] Ravel-Chapuis A., The RNA-binding protein Staufen1 is increased in DM1 skeletal muscle and promotes alternative pre-mRNA splicing. J. Cell Biol. 196, 699–712 (2012). 22431750 10.1083/jcb.201108113 PMC3308689

[15] Bondy-Chorney E., Staufen1 regulates multiple alternative splicing events either positively or negatively in DM1 indicating its role as a disease modifier. PLoS Genet. 12, e1005827 (2016). 26824521 10.1371/journal.pgen.1005827 PMC4733145

[16] Kanadia R. N., Reversal of RNA missplicing and myotonia after muscleblind overexpression in a mouse poly(CUG) model for myotonic dystrophy. Proc. Natl. Acad. Sci. U.S.A. 103, 11748–11753 (2006). 16864772 10.1073/pnas.0604970103 PMC1544241

[17] Gray S. J., Preclinical differences of intravascular AAV9 delivery to neurons and glia: A comparative study of adult mice and nonhuman primates. Mol. Ther. 19, 1058–1069 (2011). 21487395 10.1038/mt.2011.72 PMC3129805

[18] Qiao C., Muscle and heart function restoration in a limb girdle muscular dystrophy 2I (LGMD2I) mouse model by systemic FKRP gene delivery. Mol. Ther. 22, 1890–1899 (2014). 25048216 10.1038/mt.2014.141 PMC4429733

[19] Lin X., Failure of MBNL1-dependent post-natal splicing transitions in myotonic dystrophy. Hum. Mol. Genet. 15, 2087–2097 (2006). 16717059 10.1093/hmg/ddl132

[20] Huelga S. C., Integrative genome-wide analysis reveals cooperative regulation of alternative splicing by hnRNP proteins. Cell Rep. 1, 167–178 (2012). 22574288 10.1016/j.celrep.2012.02.001 PMC3345519

[21] Burd C. G., Dreyfuss G., RNA binding specificity of hnRNP A1: Significance of hnRNP A1 high-affinity binding sites in pre-mRNA splicing. EMBO J. 13, 1197–1204 (1994). 7510636 10.1002/j.1460-2075.1994.tb06369.x PMC394929

[22] Trapnell C., The dynamics and regulators of cell fate decisions are revealed by pseudotemporal ordering of single cells. Nat. Biotechnol. 32, 381–386 (2014). 24658644 10.1038/nbt.2859 PMC4122333

[23] Ladd A. N., Charlet N., Cooper T. A., The CELF family of RNA binding proteins is implicated in cell-specific and developmentally regulated alternative splicing. Mol. Cell. Biol. 21, 1285–1296 (2001). 11158314 10.1128/MCB.21.4.1285-1296.2001 PMC99581

[24] Ladd A. N., Stenberg M. G., Swanson M. S., Cooper T. A., Dynamic balance between activation and repression regulates pre-mRNA alternative splicing during heart development. Dev. Dyn. 233, 783–793 (2005). 15830352 10.1002/dvdy.20382

[25] Kumar A., Williams K. R., Szer W., Purification and domain structure of core hnRNP proteins A1 and A2 and their relationship to single-stranded DNA-binding proteins. J. Biol. Chem. 261, 11266–11273 (1986). 3733753

[26] Riva S., Mammalian single-stranded DNA binding protein UP I is derived from the hnRNP core protein A1. EMBO J. 5, 2267–2273 (1986). 3023065 10.1002/j.1460-2075.1986.tb04494.x PMC1167110

[27] Herrick G., Alberts B., Purification and physical characterization of nucleic acid helix-unwinding proteins from calf thymus. J. Biol. Chem. 251, 2124–2132 (1976). 1270425

[28] Lemieux B., A function for the hnRNP A1/A2 proteins in transcription elongation. PLoS One 10, e0126654 (2015). 26011126 10.1371/journal.pone.0126654 PMC4444011

[29] Guil S., Cáceres J. F., The multifunctional RNA-binding protein hnRNP A1 is required for processing of miR-18a. Nat. Struct. Mol. Biol. 14, 591–596 (2007). 17558416 10.1038/nsmb1250

[30] Michlewski G., Cáceres J. F., Antagonistic role of hnRNP A1 and KSRP in the regulation of let-7a biogenesis. Nat. Struct. Mol. Biol. 17, 1011–1018 (2010). 20639884 10.1038/nsmb.1874 PMC2923024

[31] Zhang Q. S., Manche L., Xu R. M., Krainer A. R., hnRNP A1 associates with telomere ends and stimulates telomerase activity. RNA 12, 1116–1128 (2006). 16603717 10.1261/rna.58806 PMC1464852

[32] Flynn R. L., TERRA and hnRNPA1 orchestrate an RPA-to-POT1 switch on telomeric single-stranded DNA. Nature 471, 532–536 (2011). 21399625 10.1038/nature09772 PMC3078637

[33] Mayeda A., Krainer A. R., Regulation of alternative pre-mRNA splicing by hnRNP A1 and splicing factor SF2. Cell 68, 365–375 (1992). 1531115 10.1016/0092-8674(92)90477-t

[34] Tavanez J. P., Madl T., Kooshapur H., Sattler M., Valcárcel J., hnRNP A1 proofreads 3′ splice site recognition by U2AF. Mol. Cell 45, 314–329 (2012). 22325350 10.1016/j.molcel.2011.11.033

[35] Akerman M., Differential connectivity of splicing activators and repressors to the human spliceosome. Genome Biol. 16, 119 (2015). 26047612 10.1186/s13059-015-0682-5 PMC4502471

[36] Venables J. P., Multiple and specific mRNA processing targets for the major human hnRNP proteins. Mol. Cell. Biol. 28, 6033–6043 (2008). 18644864 10.1128/MCB.00726-08 PMC2547008

[37] Martinez-Contreras R., Intronic binding sites for hnRNP A/B and hnRNP F/H proteins stimulate pre-mRNA splicing. PLoS Biol. 4, e21 (2006). 16396608 10.1371/journal.pbio.0040021 PMC1326234

[38] Patry C., Small interfering RNA-mediated reduction in heterogeneous nuclear ribonucleoparticule A1/A2 proteins induces apoptosis in human cancer cells but not in normal mortal cell lines. Cancer Res. 63, 7679–7688 (2003). 14633690

[39] David C. J., Manley J. L., Alternative pre-mRNA splicing regulation in cancer: Pathways and programs unhinged. Genes Dev. 24, 2343–2364 (2010). 21041405 10.1101/gad.1973010 PMC2964746

[40] Bekenstein U., Soreq H., Heterogeneous nuclear ribonucleoprotein A1 in health and neurodegenerative disease: From structural insights to post-transcriptional regulatory roles. Mol. Cell. Neurosci. 56, 436–446 (2013). 23247072 10.1016/j.mcn.2012.12.002

[41] Donev R., Newall A., Thome J., Sheer D., A role for SC35 and hnRNPA1 in the determination of amyloid precursor protein isoforms. Mol. Psychiatry 12, 681–690 (2007). 17353911 10.1038/sj.mp.4001971 PMC2684093

[42] Berson A., Cholinergic-associated loss of hnRNP-A/B in Alzheimer’s disease impairs cortical splicing and cognitive function in mice. EMBO Mol. Med. 4, 730–742 (2012). 22628224 10.1002/emmm.201100995 PMC3494073

[43] Lin Y., Protter D. S., Rosen M. K., Parker R., Formation and maturation of phase-separated liquid droplets by RNA-binding proteins. Mol. Cell 60, 208–219 (2015). 26412307 10.1016/j.molcel.2015.08.018 PMC4609299

[44] Kim H. J., Mutations in prion-like domains in hnRNPA2B1 and hnRNPA1 cause multisystem proteinopathy and ALS. Nature 495, 467–473 (2013). 23455423 10.1038/nature11922 PMC3756911

[45] Du H., Aberrant alternative splicing and extracellular matrix gene expression in mouse models of myotonic dystrophy. Nat. Struct. Mol. Biol. 17, 187–193 (2010). 20098426 10.1038/nsmb.1720 PMC2852634

[46] Batra R., Loss of MBNL leads to disruption of developmentally regulated alternative polyadenylation in RNA-mediated disease. Mol. Cell 56, 311–322 (2014). 25263597 10.1016/j.molcel.2014.08.027 PMC4224598

[47] Gudde A. E. E. G., Antisense transcription of the myotonic dystrophy locus yields low-abundant RNAs with and without (CAG)n repeat. RNA Biol. 14, 1374–1388 (2017). 28102759 10.1080/15476286.2017.1279787 PMC5711456

