# Supplementary material for: HNRNPA1-induced spliceopathy in a transgenic mouse model of myotonic dystrophy
Source: Proc Natl Acad Sci U S A. 2020 Feb 21;117(10):5472–7. doi: 10.1073/pnas.1907297117 (PMC7071875; doi:10.1073/pnas.1907297117)
Supplement: Supplementary File [file pnas.1907297117.sapp.pdf]

PNAS
www.pnas.org

Supplementary Information for

HNRNPA1-induced spliceopathy in a transgenic mouse model of myotonic dystrophy

Moyi Li[a,b,c,1,2], Yan Zhuang[a,1], Ranjan Batra[b], James D. Thomas[b], Mao Li[d], Curtis A. Nutter[b], Marina M. Scotti[b], Helmut A. Carter[b], Zhan Jun Wang[e], Xu-Sheng Huang[d], Chuan Qiang Pu[d], Maurice S. Swanson[b], Wei Xie[a,c,2]

[a]School of Life Science and Technology, The Key Laboratory of Developmental Genes and Human Disease, Southeast University, Nanjing 210096, China; [b]Department of Molecular Genetics and Microbiology, Genetics Institute and the Center for NeuroGenetics, University of Florida, College of Medicine, Gainesville, FL 32610, USA; [c]Jiangsu Co-innovation Center of Neuroregeneration, Nantong University, Nantong 226001, China [d] Department of Neurology, The General Hospital of Chinese People's Liberation Army, Beijing 100853, China; [e]Department of Neurology, Xuan Wu Hospital of Capital Medical University, Beijing 100053, China;

[1] M.L. and Y. Z. contributed equally to this work
[2]Correspondence: limoyi@seu.edu.cn; wei.xie@seu.edu.cn

This PDF file includes:

Materials and Methods
Figures S1 to S3
Legends for Movies S1 to S3
Legends for Datasets S1
SI References

www.pnas.org/cgi/doi/10.1073/pnas.1907297117

Materials and Methods

DM1 Biopsies. Muscle biopsies were obtained using procedures approved by the Institutional Review Board (The General Hospital of Chinese People's Liberation Army). All patients participating in this study provided written informed consent prior to muscle biopsy collection. This study was approved by the Institutional Review Board (The General Hospital of Chinese People's Liberation Army). Biopsies were obtained from biceps brachii or quadriceps under sterile conditions from 12 DM1 patients and 5 age-matched non-DM1 subjects as controls. Muscle samples were mounted on tetracine gel and frozen in isopentane cooled in liquid nitrogen after removing blood vessels, fat and connective tissues. The diagnosis of DM1 was based upon the clinical diagnostic criteria set by the International Consortium for Myotonic Dystrophy. Prior to protein analysis, muscle samples were sliced into 50 µm sections and ~10-20 mg of muscle sections (~10-15 sections) were lysed in 150 µl of protein isolation buffer as previously described (1) and sonicated using a Bioruptor Standard (Diagenode) to avoid volume lost and cross contamination. Protein concentration was quantified and 50 µg of protein lysates were loaded per lane for immunoblotting.

Animals. FVB WT or HSA^{LR} mice on the FVB genetic background were used for experiments. Animal procedures were performed according to guidelines established in the National Institutes of Health Guide for Care and Use of

Laboratory Mice and were approved by either the University of Florida Institutional Animal Care and Use Committee (IACUC) or Southeast University of China IACUC.

Viral Preparation and Administration. The major MBNL2 (40 kDa), HNRNPA1 (34 kDa) and CEFL1 (55kDa) isoforms in skeletal muscle were cloned, myc-tagged versions were subcloned into pTRUF12Δ and viral preparations were generated by the University of Florida Powell Gene Therapy Center as described (2). Viral injections were performed on transgenic HSA^{LR} or FVB WT mice. For systemic expression, P0-P2 pups were injected (40 µl in PBS using a 31G insulin syringe) with AAV9/GFP (2×10^{11} vg), AAV9/mycMbnl2 (5×10^{11} vg for HSA^{LR}; 2×10^{10} vg for FVB WT), AAV9/mycHnrnpA1 (2×10^{11} vg for HSA^{LR}; 2×10^{10} vg for FVB WT) or AAV9/mycCelf1 (2×10^{11} vg for HSA^{LR}; 2×10^{10} vg for FVB WT) once into the temporal vein. Four to six weeks after injection, mice were sacrificed and several muscle groups (tibialis anterior or TA, gastrocnemius, quadriceps, paraspinals) were collected for RNA and protein isolation. For intramuscular injections, the left TA was injected (1×10^{11} vg in 30 ul of PBS) as described (2). Four weeks following injection, mice were sacrificed followed by harvesting of injected and contralateral uninjected TAs. For viral infection of primary myoblast cultures, mouse skeletal muscle Hrnpa1, Celf11 and Hrnph1 isoform cDNAs were subcloned into pLenti-CMV-MCS-3FLAG-PGK-Puro and viral preparations performed by Obio Technology (Shanghai).

Immunoblotting, Muscle Functional Analysis and Histology. Dissected tissues from control, AAV9/mycMbnl2 ($5×10^{11}$ vg) and AAV9/mycHnrnpA1 injected mice were homogenized, sonicated and centrifuged as described previously (2, 3). Proteins (50µg /lane) were detected by immunoblotting using anti-MBNL2 mouse monoclonal (mAb) 3B4 (Santa Cruz Biotechnology, 1:500), anti-MBNL1 rabbit polyclonal Ab(pAb) A2764 (1:1000) or rabbit pAb ab45899 (Abcam, 1:1000), anti-HNRNPA1 mAb 4B10 (Santa Cruz Biotechnology, 1:1000), anti-HNRNPA2B1 mAb DP3B3 (Abcam, 1;1000), anti-HNRNPH1 pAb ab10347 (Abcam, 1:1000), anti-CELF1 mAb 3B1 (Abcam, 1:1000), anti-myc mAb 9E11 (Novus,1:1000), anti-ELAVL1 mAb 3A2 (1:1000) (4), anti-Gapdh mAb 6C5 (Abcam,1:1000) and HRP- conjugated anti-mouse secondary antibody followed by ECL (GE Healthcare). For grip strength, mice (n=6) were assessed for forelimb grip strength using a grip strength meter (Columbia Instruments). For histology, frozen tissue sections (10 µm) of systemically injected mice, or TA muscle for IM injections, were prepared for hematoxylin and eosin (H&E) staining. Western blots were quantified using ImageJ and one-tailed unpaired t test with Welch's correction was used for statistical analysis. For NMJ imaging, muscle fibers were dissected and stained as described previously (5).

Primary Myoblast Culturing and Lenti-viral Transduction. Primary myoblasts were isolated, purified and transduced by viruses based on previously described protocols (6-8). Briefly, hindlimb muscle tissue of three to

five neonatal (P0-P6) mice was minced using sterile razor blades and digested in DMEM media containing 0.125% trypsin for 1 h at 37°C. The digested mixture was triturated using a disposable Pasteur transfer pipette and filtered through a 70-µm cell strainer. After centrifugation, the cell pellet was resuspended in myoblast growth medium (Ham's F10, 20% FBS, 1% penicillin/streptomycin, 5-10ng/µl bFGF), and cells were grown on 10% matrigel-coated tissue culture dishes at 37°C and 5% CO_2. Fibroblasts were depleted from myoblasts by 3-4 rounds of pre-plating and seeding cycles. Purified myoblasts >70% confluency were transduced by lenti viruses and were allowed to undergo in vitro myogenic differentiation into myotubes following plating on 10% matrigel in differentiation medium (DMEM, 5% horse serum, 1% penicillin/streptomycin) for 3 days. Puromycin (2 mg/ml) selection for lenti-transduce myofibers was performed for 4 days followed by RNA and protein isolation 1 week following the induction of differentiation.

Splicing Analysis. For detecting alternative splicing in vivo, cDNAs were synthesized by reverse transcription using 5 µg of total RNA from harvested tissues and SuperScript III RNaseH-RT (Invitrogen). Subsequent radioactive PCR was performed as described (5) using 5% of the total RT reaction as a template. For splicing assays in myoblast cultures, non-radioactive PCR was performed and agarose gel images were quantified using ImageJ according to user guide.

CLIP-seq and RNA-seq. HITS-CLIP was performed as described previously (4, 9) with the following modifications. Quadriceps muscles were dissected from 12 week-old *HSA*^{LR} mice injected with rAAV2/9-HnrnpA1/34kDa (n=3), snap frozen in liquid nitrogen, ground to a fine powder and crosslinked with UV-light in a Stratalinker 1800 (Stratagene). Crosslinked samples were treated with RNase A (55 U/ml and 0.55 U/ml for high and low RNase, respectively) to generate RNA tags, RNA-protein complexes were purified with anti-HNRNPA1 mAb 4B10 (5 µg) and cDNA libraries were generated using the RNA linkers and primers described for Ago CLIP (10). All sequencing was performed on the Illumina platform and raw reads obtained from the Illumina pipeline include a 4 nt barcode (3 random positions and a G at the 4th position) at the 5' end followed by the CLIP tag and 3' adaptor sequence. Datasets were filtered to remove low quality reads by requiring a minimum score of 20 in barcode positions and an average score of 20 in the following 25 nucleotides. The filtered reads were then aligned to the mouse reference genome (mm9) and potential PCR duplicates, as judged from the starting position of genomic mapping and the barcode sequences, were removed to identify unique CLIP tags as described previously (4, 9). Peaks were identified using tag2peak function in the CIMS software suite. The tags were also clustered if the gap between them was less than 1 bp using the tag2cluster function in the CIMS software suite. For CIMS analysis, we estimated deletions using BWA in Galaxy. Paired-end RNA-seq datasets from myoblast, myocyte, nascent myotube, and mature myotube (n=3) samples were obtained from the Gene

Expression Omnibus (GSE52529) and paired-end RNA-seq data (n=3) was retrieved from DMseq.org. Reads were pseudo- aligned to the human transcriptome (Gencode 24) and transcript abundance generated using Kallisto (11). Transcript per million (tpm) values were summed to generate gene expression values from the three biological replicates.

Muscle Injury/Regeneration. To recapitulate muscle regeneration process, muscle injury was made on adult mice as described (4) by Notexin (Latoxan) injection. And injected muscle samples were collected for immunoblotting at different time points.

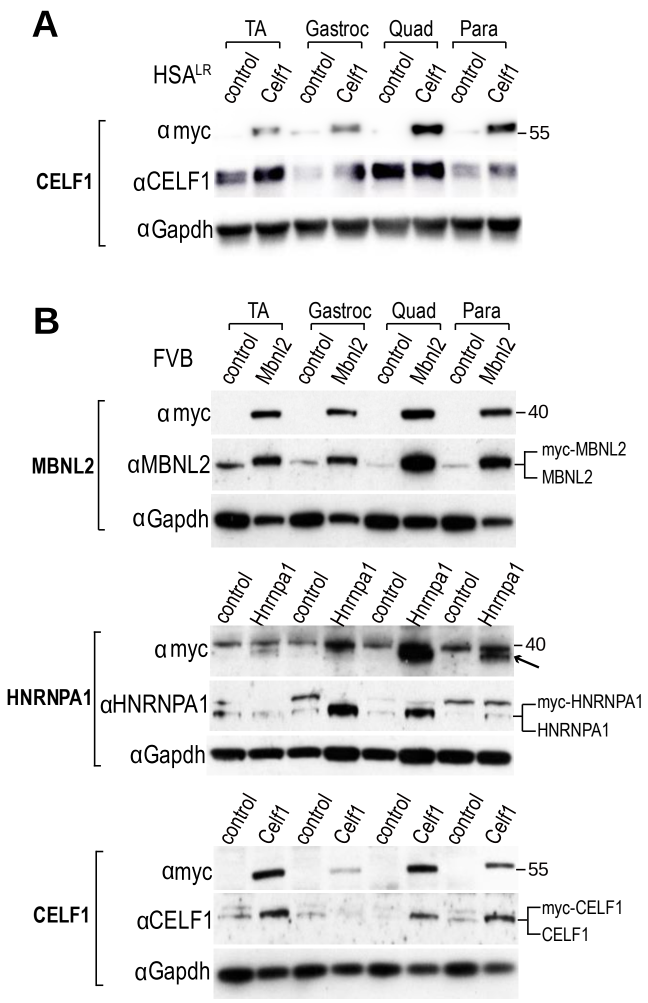

Figure S1. AAV9-mediated systemic overexpression of CELF1 in *HSA*^{LR} and overexpression of MBNL2, HNRNPA1 and CELF1 in FVB WT mice. The overexpression of all three transgenes in FVB WT displayed similarly varied expression levels in different muscles compared to the patterns in transduced *HSA*^{LR} mice. (A) CELF1 transgene expression patterns in different muscles of rAAV2/9-mycCelf1 injected *HSA*^{LR}. (B) Transgene expression patterns in different muscles of rAAV2/9-mycMbnl2, rAAV2/9-mycHnrnpA1 or rAAV2/9-mycCelf1 injected FVB WT mice. rAAV9 (2×10^{10} vg) viruses were injected into FVB WT P0 mice (control, PBS-injected FVB WT mice; arrow, myc-HNRNPA1). Note that the MBNL2 isoform (355aa) used in this FVB WT experiment is a shorter isoform in muscle lacking exon 8 (54bp).

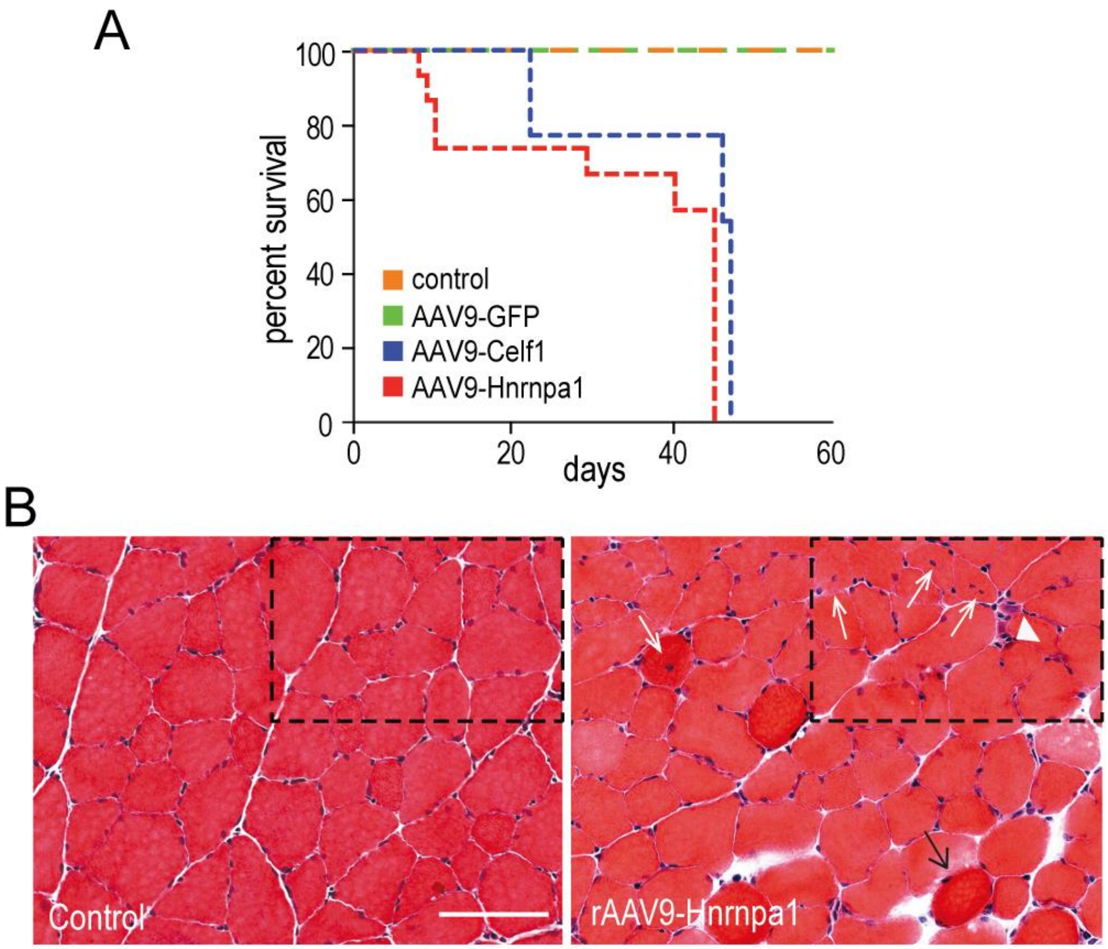

Figure S2. AAV9-mediated systemic overexpression of HNRNPA1 in *HSA*^{LR} leads to reduced lifespan and earlier onset of DM-like myopathic features. (A) *HSA*^{LR} P0-P2 mice were injected (IV) with either PBS (control, n=5), AAV9-GFP (n=5), AAV9-HnrnpA1 (n=14), or AAV9-Celf1 (n=8). HNRNPA1 or CELF1 overexpression led to reductions in lifespan compared to PBS or GFP injected mice. (B) Representative quadriceps cross-section images of HNRNPA1 (rAAV9-Hnrnpa1) injected *HSA*^{LR} compared to uninjected ones at 4-6 weeks of age. *HSA*^{LR} injected with rAAV9-Hnrnpa1 displayed increased centralized myonuclei (white arrows), atrophic (white arrowhead) myofibers, split fibers and sporadic ring fibers (black arrow). The regions within rectangles with black dashed lines are further magnified and displayed in Figure 1*D*.

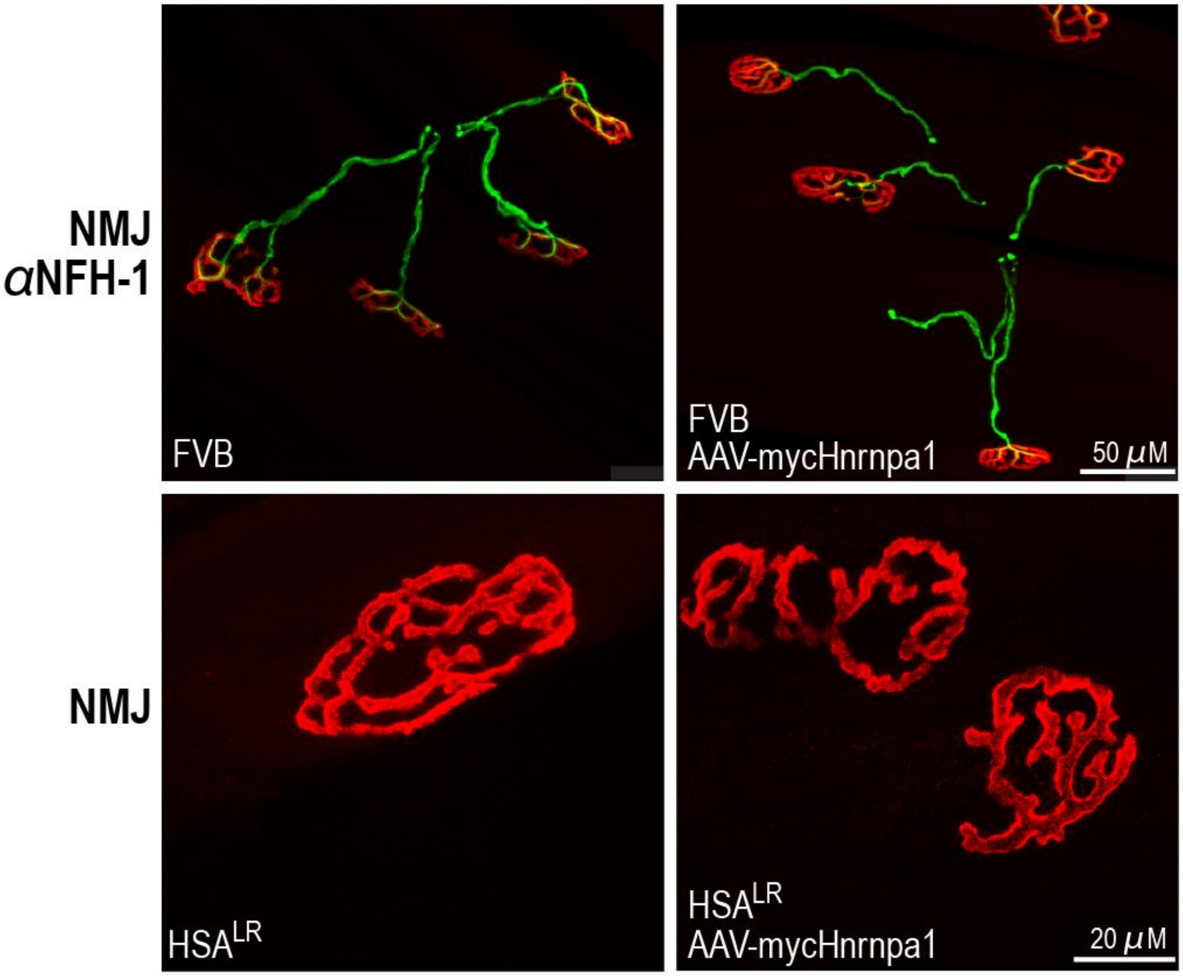

Figure S3. NMJ morphology is not markedly affected by HNRNPA1 overexpression in either FVB or *HSA*^{LR} mice. AAV9-Hhrnpa1 was injected into the temporal vein of P2 mice. TA myofibers were dissected from P30 injected and uninjected mice and stained with α- bungarotoxin for NMJ endplates and immunostained with NFH-1 antibody (aNFH-1) for innervating neurons.

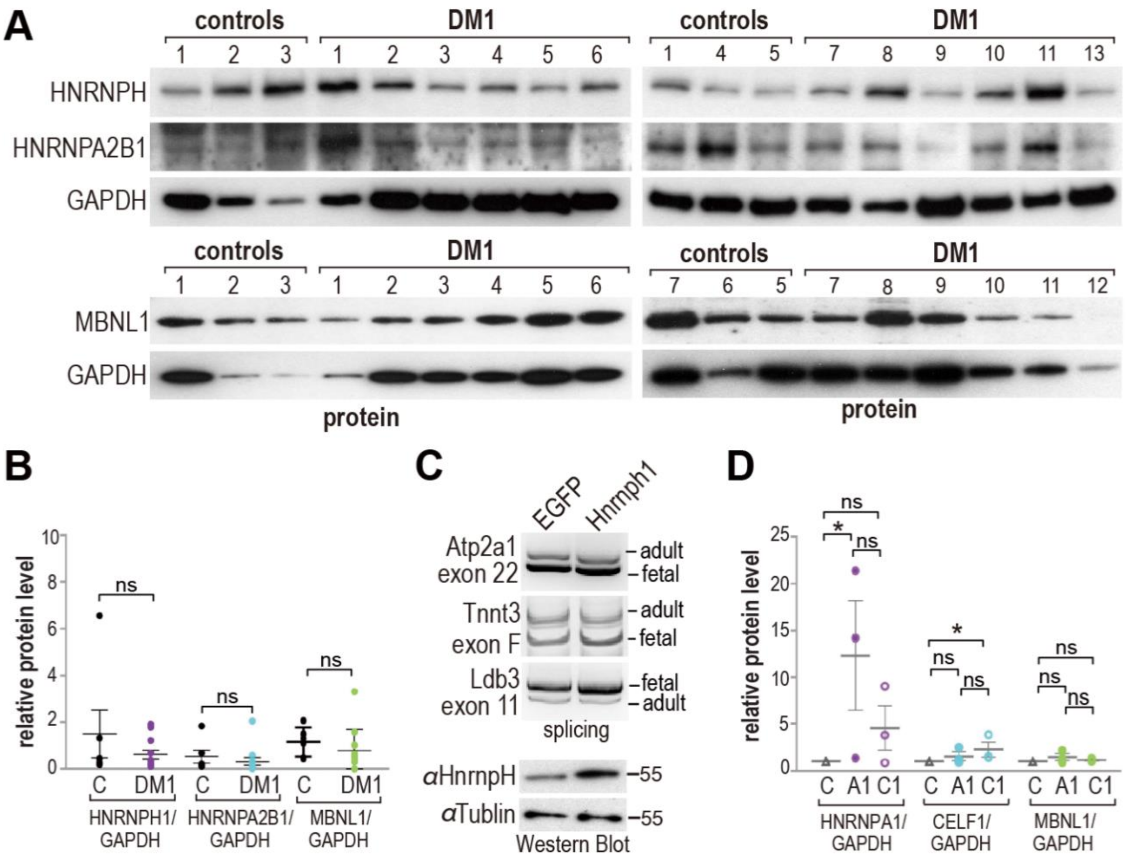

Figure S4. Expression levels of HNRNPH1, HNRNPA2B1 and MBNL1 in DM1 samples and splicing effects of HNRNPH1 on DM1 targets in myoblast primary cultures. HNRNPH1, HNRNPA2B1 and MBNL1 are not significantly changed in DM1 biopsies. (*A*) Immunoblots of control (n=5) and DM1 (n=12) muscle biopsy samples (GAPDH, loading control). (*B*) Quantitative analysis of relative protein levels (HNRNPH1/GAPDH, HNRNPA2B1/GAPDH and MBNL1/GAPDH) between control and DM1 muscle biopsies. P-values were calculated using unpaired one-tailed Student's t-test. Data are SEM (ns: not significant). (*C*) HNRNPH1 overexpression in cultured myoblasts does not lead to an increase of fetal exon inclusion in differentiated myotubes. (*D*) Quantitative analysis of relative protein levels (HNRNPA1/GAPDH, HNRNPCELF1/GAPDH and MBNL1/GAPDH) between control (C, n=3), AAV/HnrnpA1 (A1, n=3) and AAV/Celf1 (C1, n=3) transduced myofibers. P-values were calculated using a one-way ANOVA with Dunnet's post-hoc test. Data are SEM (*, $P < 0.05$; ns: not significant).

Other supplementary materials for this manuscript include the following:

Movie S1 (separate file). Cage mobility and myotonia (induced by hindlimb pinching) of *HSA*^{LR} FVB mice that were not injected with rAAV. Note the myotonia is transient following induction.

Movie S2 (separate file). Clasping, partial limb paralysis and myotonia of *HSA*^{LR} FVB mice injected with AAV9/2-mycHnrnpa1 to overexpress the HNRNPA1 protein.

Movie S3 (separate file). Abnormal gait patterns (DigiGait analysis) of AAV9/2-mycHnrnpa1 injected *HSA*^{LR} mice compared to control *HSA*^{LR} uninjected mice.

Dataset S1(separate file). Spreadsheets for HNRNPA1 HITS-CLIP data analysis, RNA-seq data analysis of myoblasts and differentiating myofibers and human muscle biopsy sample information. (A) Genomic Distribution of HNRNPA1 HITS-CLIP Reads; (B) HNRNPA1 binding peaks as reported by tag2peak program in CIMS (mm9); (C) HNRNPA1 binding clusters as reported by tag2cluster program in CIMS (mm9); (D) Overlapping HNRNPA1 CLIP-Peaks with DM1 related (*HSA*^{LR}) AS events; (E) Analysis for reported RNA-seq data (DMseq.org) of interested RNA binding proteins in different muscle cells including myoblasts, myocytes, nascent myotubes and mature myotubes; (F) Human muscle biopsy sample information and genotype

References

1. Iyer RR, Pluciennik A, Napierala M, & Wells RD (2015) DNA triplet repeat expansion and mismatch repair. *Annu Rev Biochem* 84:199-226.
2. Kanadia RN*, et al.* (2006) Reversal of RNA missplicing and myotonia after muscleblind overexpression in a mouse poly(CUG) model for myotonic dystrophy. *Proc Natl Acad Sci U S A* 103(31):11748-11753.
3. Charizanis K*, et al.* (2012) Muscleblind-like 2-mediated alternative splicing in the developing brain and dysregulation in myotonic dystrophy. *Neuron* 75(3):437-450.
4. Batra R*, et al.* (2014) Loss of MBNL leads to disruption of developmentally regulated alternative polyadenylation in RNA-mediated disease. *Mol Cell* 56(2):311-322.
5. Lee KY*, et al.* (2013) Compound loss of muscleblind-like function in myotonic dystrophy. *EMBO Mol Med* 5(12):1887-1900.
6. Thomas JD*, et al.* (2017) Disrupted prenatal RNA processing and myogenesis in congenital myotonic dystrophy. *Genes Dev* 31(11):1122-1133.

7. Hindi L, McMillan JD, Afroze D, Hindi SM, & Kumar A (2017) Isolation, Culturing, and Differentiation of Primary Myoblasts from Skeletal Muscle of Adult Mice. *Bio Protoc* 7(9).
8. Shahini A, *et al.* (2018) Efficient and high yield isolation of myoblasts from skeletal muscle. *Stem Cell Res* 30:122-129.
9. Goodwin M, *et al.* (2015) MBNL sequestration by toxic RNAs and RNA misprocessing in the Myotonic Dystrophy brain. *Cell Rep* 12(7):1159-1168.
10. Moore MJ, *et al.* (2014) Mapping Argonaute and conventional RNA-binding protein interactions with RNA at single-nucleotide resolution using HITS-CLIP and CIMS analysis. *Nat Protoc* 9(2):263-293.
11. Bray NL, Pimentel H, Melsted P, & Pachter L (2016) Near-optimal probabilistic RNA-seq quantification. *Nat Biotechnol* 34(5):525-527.
